# Effect of Imitation Phenomenon on Two-Lane Traffic Safety in Fog Weather

**DOI:** 10.3390/ijerph16193709

**Published:** 2019-10-01

**Authors:** Jinhua Tan, Li Gong, Xuqian Qin

**Affiliations:** School of Information and Safety Engineering, Zhongnan University of Economics and Law, Wuhan 430073, China; gongli@zuel.edu.cn (L.G.); qinxq@zuel.edu.cn (X.Q.)

**Keywords:** traffic safety, imitation phenomenon, two-lane car-following model, fog weather

## Abstract

A neighboring lane’s vehicles are potentially important influence factors of traffic safety. In fog weather, drivers will automatically imitate the behaviors demonstrated by other vehicles in the neighboring lane. To illustrate the effect of the imitation phenomenon on traffic safety, this paper develops an extended two-lane car-following model in fog weather. Numerical simulations are carried out to study the effect of imitation on multiple-vehicle collision induced by a sudden stop, as well as perturbation propagation when a small perturbation is added to the uniform traffic flow. The results indicate that the number of collisions depends on the influence coefficient of neighboring lane’s vehicles, sensitivity, headway and initial velocity. Furthermore, the number of crumpled vehicles decreases when the imitation phenomenon is taken into account. In addition, lower vehicular velocity in the neighboring lane can reduce the magnitude of acceleration and fluctuation of headway. The perturbation can be absorbed under certain given conditions regarding the imitation phenomenon. Therefore, traffic safety can be improved by considering the effect of the imitation phenomenon on two-lane traffic flow in fog weather. The findings in this study can provide a theoretical reference for the development of multi-lane intermittent release measures in fog weather.

## 1. Introduction

Traffic accidents are a significant threat to public health in every country [1]. Fog is a dangerous weather condition, which could contribute to a considerable number of traffic accidents. In the United States, nearly 600 people are killed and 16300 people are injured annually in fog-related crashes [2]. In China, about 25 percent of traffic accidents on the Shanghai-Nanjing freeway are fog-related [3]. Traffic safety problems in fog have received much attention recently [4,5,6,7,8,9,10,11,12]. In foggy weather, drivers demonstrate the risk behavior of shortening the space headway in one-lane and two-lane traffic systems due to cognitive limitations, resulting in an increase in the probability of rear-end crashes [4,5,6,7,8,9].

Microscopic traffic models can describe traffic behaviors, where car-following models have received considerable attention [10,11,12,13,14,15,16,17,18,19,20,21,22,23,24,25,26]. In order to make effective policies to solve the complex problems in traffic safety management, various models have been proposed with a focus on fog weather in recent years. In China, freeways are frequently closed in heavy fog for safety purposes, which seriously affects travelling. Different from the closure of freeways, intermittent release measures mean that bureaus of freeway administration release a limited number of small vehicles within a specified time interval, which has been seen to improve traffic safety in some regions [11]. In order to describe and explore the effect of intermittent release measures on traffic, some cellular automation models were proposed [10,11,12]. Results indicated that intermittent release measures could reduce car accident probability as well as exhaust emission. In addition, with consideration of drivers’ speeding up unintentionally and misjudgment of space headway, Tan [26] set up a car-following model including risk illusions in fog weather (RIFM). The simulation results illustrated that risk illusions have disadvantageous impacts on traffic flow stability, velocity, acceleration and headway fluctuation frequency in a one-lane traffic system. Furthermore, the risk of vehicle crashes has aroused wide concern [27,28,29,30,31,32,33,34], which can also be investigated on the basis of car-following model [35,36,37,38,39,40]. Especially, Sugiyama and Nagatani derived the collision criterion to study the factors affecting multiple-vehicle collisions induced by a sudden stop [35].

Ponnu and Coifman [41,42] indicated that car-following behaviors not only depended on the vehicles in the same lane, but also depended on the relative speed to the adjacent lane. Duan et al. investigated whether the headway distance was affected by the distance demonstrated by other vehicles in the neighboring lane and found that both novice and experienced drivers’ behaviors were affected by vehicles in the neighboring lane [43]. This phenomenon was called automatic imitation because the drivers were rarely aware of the influence of the neighboring lane’s vehicles. A driving simulator experiment was conducted on a one-way two-lane freeway [5]. In the experiment, driving behaviors of overtaking and lane changing were prohibited due to fog weather. The results confirmed that drivers’ behaviors and psychological characteristics in fog were influenced by the vehicles in the neighboring lane. Therefore, vehicles in the neighboring lane are potentially important influence factors of traffic safety.

However, the effect of driving behaviors influenced by the neighboring lane’s vehicles on traffic safety is infrequently discussed in the existing literature of the car-following model. To enrich the understanding of the influence of the neighboring lane’s vehicles on traffic safety as well as its mechanism, this paper develops an extended two-lane car-following model accounting for the imitation phenomenon from neighboring lane’s vehicles in fog weather. Numerical simulations of the proposed model are carried out to explore the effect of this imitation phenomenon on multiple-vehicle collision. Moreover, the propagation of small perturbation is analyzed to explore its influence mechanism on traffic safety. The findings of this study may provide a reference for the development and implementation of multi-lane intermittent release measures in fog weather. Compared to the single-lane release measures, the multi-lane intermittent release measures are considered to have a more positive effect on traffic accident control and prevention. The remainder of this paper is organized as follows. Section 2 introduces the new model and the numerical methods in detail. In Section 3, the numerical results are described. The findings, strengths and limitations of this work are discussed in Section 4, followed by conclusions in Section 5.

## 2. Methodology

### 2.1. Model

#### 2.1.1. Imitation Phenomenon

Tan [5] investigated driver’s behaviors on a two-lane freeway in fog weather using a driving simulator experiment combined with the use of a questionnaire. It has been found from interviews with some experienced drivers that driving one vehicle alone in fog can often be very difficult, while the appearance of vehicles in other lanes may have a certain influence on driving behaviors. Some drivers may choose to drive parallel to the vehicles in a neighboring lane to reduce the anxiety of driving alone. Three groups of driving simulator experiments were conducted to study the influence of the neighboring lane’s vehicles on driving behaviors in fog weather. The scenario environment provided by the driving simulator included a one-way, two-lane freeway, vehicles and weather (clear and fog). Drivers were asked to drive at a comfortable speed in Lane 1 during the experiment.

In the experiments, there were no vehicles in Lane 2 (neighboring lane) in the scenario for Group 1 (neighboring lane with no vehicles, NLNV). There were vehicles at a low speed of 20 km/h in Lane 2 for Group 2 (neighboring lane with low speed vehicles, NLLS). For Group 3, the speed of the vehicles in Lane 2 was 60 km/h (neighboring lane with high speed vehicles, NLHS). The average speed of vehicles in Lane 1 under different visibility conditions for the three groups is shown in Table 1.

From Table 1, it is clear that, under different visibility conditions, the average speed of Group 2 (NLLS) is always lower than Group 1 (NLNV), while the average speed of Group 3 (NLHS) is always higher than Group 1 in fog weather. The results mean that drivers tend to drive at a slow speed if the speed of neighboring lane’s vehicles is relatively slow, and vice versa. In other words, drivers tend to imitate the speed of the neighboring lane’s vehicles in fog weather.

#### 2.1.2. Two-Lane Car-Following Model

To explore the impact of driving behaviors in fog weather on single-lane traffic flow, Tan [26] proposed a car-following model including risk illusions (RIFM) as below:

(1) $$\frac{d^{2}x_{n}(t)}{dt^{2}}=\alpha \left[V_{f}(\Delta x_{n}(t))-v_{n}(t)\right]+\beta \Delta v_{n}(t)+\lambda F(\Delta x_{n}(t)),$$

where parameter *α* denotes a driver’s sensitivity, *β* denotes the coefficient of the velocity difference, *λ* is a sensitivity parameter, $v_{n}(t)$ and $\Delta x_{n}(t)=x_{n+1}(t)-x_{n}(t)$ represent the speed and space headway of vehicle n at time t respectively. The function $F(\Delta x_{n}(t))=\tanh(\Delta x_{n}(t)-d_{f})$ is considered to investigate the behavior of drivers speeding up unintentionally when the space headway is beyond the comfortable range $d_{f}$. The optimal velocity function $V_{f}(\Delta x_{n}(t))$ is given as follows [26]:

(2) $$V_{f}(\Delta x_{n}(t))=\kappa \left\{V_{1}+V_{2}\tanh\left[C_{1}(\gamma \Delta x_{n}(t)-l_{c})-C_{2}\right]\right\},$$

where parameter *κ* is adopted to adjust the speed limit in low visibility, coefficient *γ* is adopted to describe the misjudgment of driving space headway. $l_{c}$ is the vehicle length, and $l_{c}=5\;m$. $V_{1}$, $V_{2}$, $C_{1}$ and $C_{2}$ are parameters, and their calibrated values are $C_{1}=0.13{\;m}^{-1}$, $C_{2}=1.57$, $V_{1}=6.75\;m/s$ and $V_{2}=7.91\;m/s$ [17].

**Figure 1:**
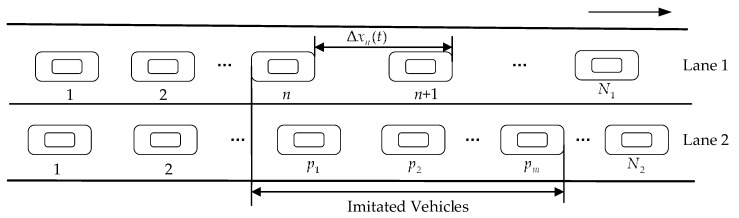
Scheme of two-lane car-following in fog weather. The single arrow in the figure shows the driving direction.

Figure 1 shows the scheme of two-lane car-following model in fog weather. Apart from the influence factors considered in the RIFM, additional factor $G(\Delta v_{n,i,\mathrm{other}}(t))$ in lane i is taken into account to describe the imitation phenomenon in this study. It is assumed that the drivers’ behaviors affected by the neighboring lane’s vehicles in both lanes are the same. The function $G(\Delta v_{n,i,\mathrm{other}}(t))$ in each lane can be simply formulated by

(3) $$\left\{ \begin{array}{l} \begin{array}{ll} G(\Delta v_{n,i,\mathrm{other}}(t))= & p_{1}(v_{n,i,1,\mathrm{other}}(t)-v_{n,i}(t))+p_{2}(v_{n,i,2,\mathrm{other}}(t)-v_{n,i}(t))\\ & +\cdots +p_{m}(v_{n,i,m,\mathrm{other}}(t)-v_{n,i}(t)),\;m>0 \end{array}\\ p_{1}+p_{2}+\cdots +p_{m}=1 \end{array}\right.,$$

where *m* is the number of preceding cars within a certain distance range $d_{\mathrm{other}}$ in the neighboring lane, $p_{1}$, $p_{2}$, … and $p_{m}$ represent the weight factors for the relative speed, $v_{n,i}(t)$ is the speed of vehicle n at time t in lane i, $\Delta v_{n,i,\mathrm{other}}(t)$ is the average velocity difference between the subject vehicle n and any other potential vehicles in a neighboring lane.

According to the above analysis of the imitation phenomenon, we can obtain a new two-lane car-following model in fog weather as follows:

(4) $$\frac{d^{2}x_{n,i}(t)}{dt^{2}}=\alpha \left[V_{f}(\Delta x_{n,i}(t))-v_{n,i}(t)\right]+\beta \Delta v_{n,i}(t)+\lambda F(\Delta x_{n,i}(t))+\eta G(\Delta v_{n,i,\mathrm{other}}(t)),$$

where $x_{n,i}(t)$ is the position of vehicle n at time t in lane i, the space headway $\Delta x_{n,i}(t)=x_{n+1,i}(t)-x_{n,i}(t)$, the velocity difference $\Delta v_{n,i}(t)=v_{n+1,i}(t)-v_{n,i}(t)$, and *η* denotes the coefficient of the imitation phenomenon.

### 2.2. Numerical Simulations

Based on the proposed two-lane car-following model, the effect of the imitation phenomenon on traffic safety in fog weather is explored using numerical methods. The numerical simulations, including the simulations of multiple-vehicle collision and small perturbation propagation, are carried out under the periodic boundary condition using Matlab R2016a. Initially, vehicles are uniformly distributed on the two-lane highway with length *L*. $N_{1}$ and $N_{2}$ respectively stand for the number of vehicles in Lane 1 and Lane 2. The parameters of $\kappa_{1}$ and $\kappa_{2}$ are adopted to adjust the speed limits in Lane 1 and Lane 2 in fog weather respectively. In this study, we simply take $p_{1}=p_{2}=\cdots =p_{m}$ and $d_{f}=d_{\mathrm{other}}=30\;m$ [26].

#### 2.2.1. Collision Criterion

Multiple-vehicle collision is a common traffic accident in fog weather. This study investigates whether or not the imitation phenomenon can influence the incidence of collisions through numerical simulations.

Sugiyama and Nagatani [35] proposed the collision criterion based on the optimal velocity function as described in Equation (5).

(5) $$V(\Delta x_{n}(t))=v_{\max}\left[\tanh(\Delta x_{n}(t)-x_{c})+\tanh(x_{c})\right]/2,$$

where $v_{\max}$ represents the maximal speed and $x_{c}$ represents the position of turning point. Vehicle n comes into collision with the vehicle in front when the space distance between them reaches zero, and the velocity $v_{n}(t)$ approaches a constant C (C>0) [35]:

(6) $$\Delta x_{n}(t)\to 0,\;\mathrm{and}\;v_{n}(t)\to C.$$

The mathematical expression of the collision between vehicle n and the vehicle in front can be expressed as follows [35]:

(7) $$\frac{v_{n}(t)}{\Delta x_{n}(t)}\to \infty ,\;t\to \infty .$$

In our study, the vehicle length is $l_{c}$. Therefore, the collision criterion can be expressed as Equations (8) and (9) correspondingly.

(8) $$\Delta x_{n}(t)-l_{c}\to 0,\;\mathrm{and}\;v_{n}(t)\to C,$$

(9) $$\frac{v_{n}(t)}{\Delta x_{n}(t)-l_{c}}\to \infty ,\;t\to \infty .$$

The collision criterion is introduced into numerical simulations to determine whether a rear-end collision will occur. The parameters for the numerical simulations of multiple-vehicle collision are defined as follows: $\kappa_{1}=\kappa_{2}=0.8$, $N_{2}=100$, L=800 m, λ=0.1, γ=1.1, time step Δt=0.001 s [40]. The initial headway in Lane 2 is $L/N_{2}$.

#### 2.2.2. Perturbation Analysis

In real traffic, perturbations such as sudden acceleration and abrupt deceleration may lead to safety problems. Perturbation analysis is a common method for studying traffic problems [44]. If a small perturbation can be absorbed during the propagation process, the traffic flow will maintain stability. Conversely, if a small perturbation propagates upstream along the traffic flow, the traffic flow may evolve into unstable flow ultimately, which is more likely to result in crashes. The perturbation analysis method is widely used to test the effects of various factors on traffic [16,45,46,47].

In order to further explore the effect mechanisms of the imitation phenomenon in fog weather, we studied the effect of small perturbations on traffic factors such as velocity, acceleration and headway under different *η* and different velocities in the neighboring lane. These factors are important indicators for traffic stability and safety. For instance, higher velocity and shorter headway correspond to more dangerous traffic in fog weather. The severe fluctuations of acceleration represent the possibility of frequent abrupt acceleration or deceleration, which is harmful to traffic safety. Similarly, the amplitudes of velocity and headway fluctuations can also reflect the stability and safety of traffic flow. Inserting a small perturbation $\Delta x_{n}(0)$ to the uniform traffic flow is a general approach to explore the stability of a traffic system [18,48,49]. Accordingly, the small perturbation added to the uniform traffic flow is the headway deviation $\Delta x_{1}(0)=5\;m$ in this study. The parameters for the simulations of perturbation propagation are set as follows: γ=1.1, λ=0.1, $N_{1}=N_{2}=100$, L=1500 m; the length of each time step is 0.1 s [26].

## 3. Results

### 3.1. Multiple-Vehicle Collision

#### 3.1.1. Multiple-Vehicle Collision under Different *η*

**Figure 2:**
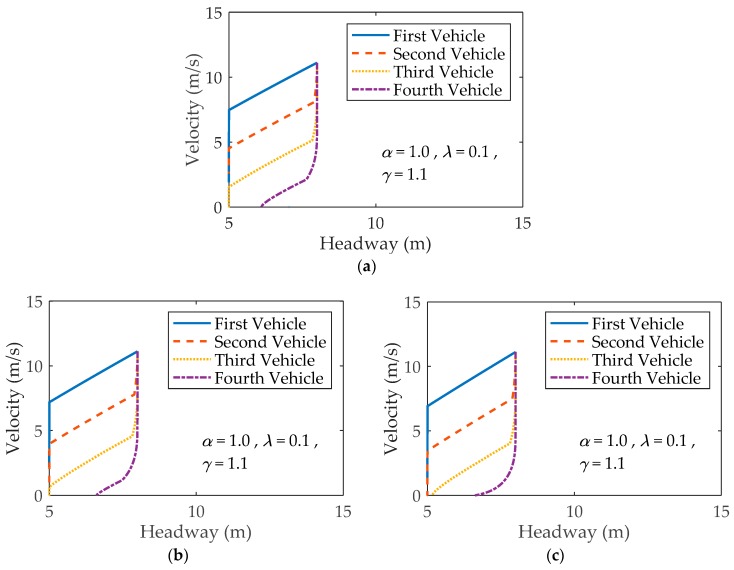
Four trajectories for four vehicles behind the suddenly stopped vehicle under different *η*: (**a**) η=0; (**b**) η=0.1; (**c**) η=0.2.

As shown in Figure 2, the velocity–headway diagrams are drawn to study how the velocity and headway vary with time when a vehicle in Lane 1 stops suddenly at t=0 [35]. Patterns (a), (b) and (c) in Figure 2 stand for the trajectories of vehicles moving behind the suddenly stopped vehicle under different parameter *η*. The trajectories are obtained by plotting the velocity against the headway at time *t*, where α=1.0, $N_{1}=100$, the initial velocity in Lane 1 is $v_{ini}=40\;\mathrm{km}/h$, and the initial velocity in Lane 2 is determined by Equation (2). Then, as time goes on, the speeds in the two lanes are both adjusted using the model in Equation (4).

Figure 2a depicts the trajectories for the first, second, third and fourth vehicles behind the stopped vehicle without consideration of vehicles in the neighboring lane. For the first vehicle, as its headway reduces to 5 m (the vehicle length), which means the space distance between it and the stopped vehicle reduces to zero, its velocity is greater than zero. According to Equation (9), a collision occurs. Specifically, the first vehicle collides with the stopped vehicle with a finite velocity (residual velocity). Similarly, the second and third vehicles hit their preceding vehicles. For the fourth vehicle, its velocity reduces to zero when the headway is larger than 5 m. Therefore, the fourth vehicle stops with a finite headway and does not collide with the vehicle in front. From Figure 2b, we can also observe that there are three vehicles colliding with their preceding vehicles when η=0.1. However, the residual velocity of the third vehicle is less than that of the third vehicle in Figure 2a, which means the severity of a rear-end collision is diminished. Figure 2c shows that there are only two vehicles colliding with their preceding vehicles when η=0.2. It is revealed that the imitation phenomenon is able to reduce the risk of collision in fog weather.

**Figure 3:**
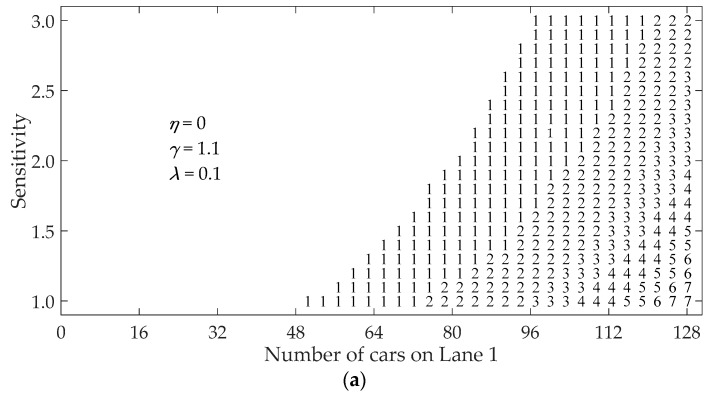

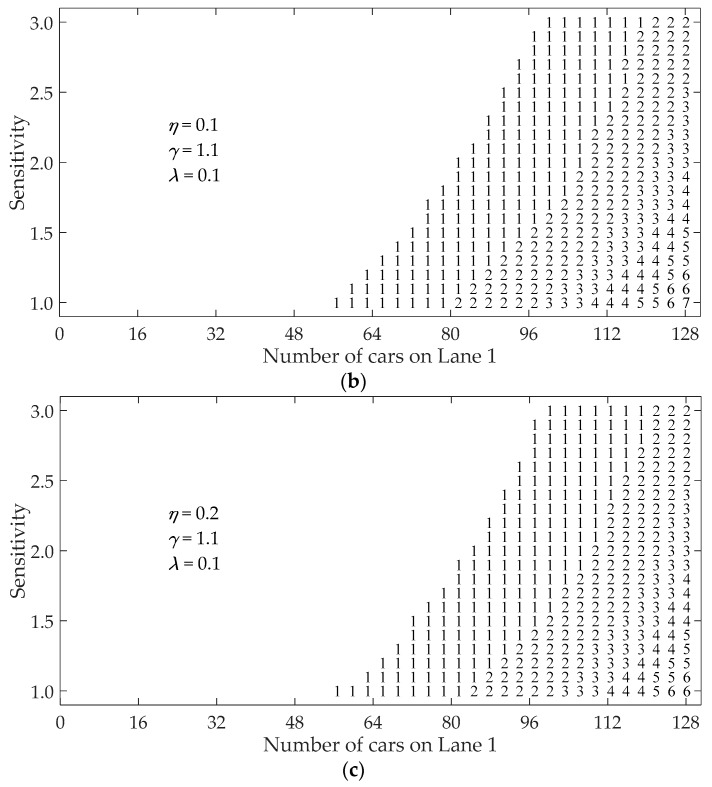
Region map (phase diagram) under different *η*: (**a**) η=0; (**b**) η=0.1; (**c**) η=0.2. The numeral characters represent the number of crumpled vehicles, and the non-marked region represents no collision.

Figure 3 illustrates the region map (phase diagram) for the multiple-vehicle collision when a vehicle in Lane 1 stops suddenly in traffic flow with initial velocity $v_{ini}=40\;\mathrm{km}/h$. The region map is drawn by varying both the sensitivity of the drivers and the number of vehicles in Lane 1. If the criterion in Equation (9) is satisfied, a collision occurs. 

The non-marked (blank) area in Figure 3 represents no collision in the traffic system. The numeral characters represent the number of crumpled vehicles in Lane 1 induced by a sudden stop. Patterns (a) and (b) in Figure 3 demonstrate that the non-marked region extends and the number of crumpled vehicles decreases with consideration of the vehicles in the neighboring lane. Furthermore, by comparing pattern (b) with pattern (c) in Figure 3, we can see that the collision region shrinks and the number of the crumpled vehicles reduces as coefficient *η* increases.

As we know, the number of crumpled vehicles decreases with the increase of driver’s sensitivity and the reduction of initial headway in clear weather [35,36]. From Figure 3, it is obvious that, in addition to sensitivity and initial headway, the occurrence of multiple-vehicle collision becomes low with consideration of the vehicles in the neighboring lane in fog weather.

#### 3.1.2. Multiple-Vehicle Collision under Different Initial Velocity $v_{ini}$

In general, the speed of a vehicle should be no more than 40 km/h in low-visibility conditions [5]. Therefore, typical values of the initial velocity below 40 km/h are tested to show the influences of $v_{ini}$ on multiple-vehicle collision in the proposed model.

**Figure 4:**
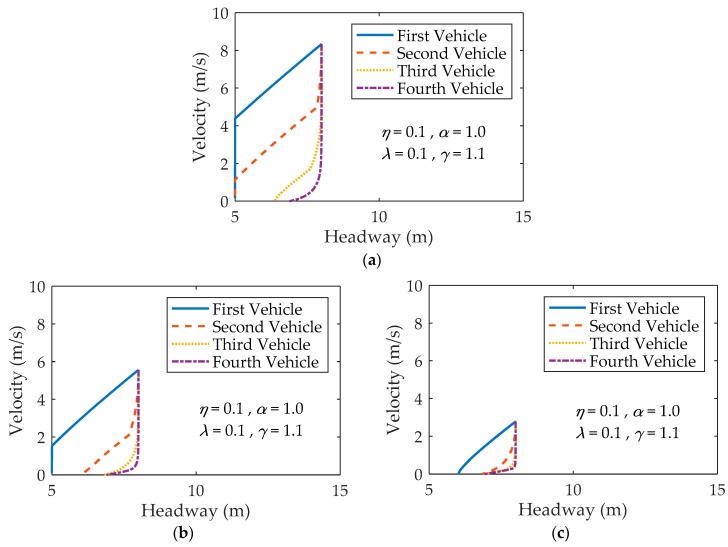
Four trajectories for four vehicles behind the suddenly stopped vehicle under different $v_{ini}$: (**a**) $v_{ini}=30\;\mathrm{km}/h$; (**b**) $v_{ini}=20\;\mathrm{km}/h$; (**c**) $v_{ini}=10\;\mathrm{km}/h$.

Three subfigures in Figure 4 show the trajectories of four vehicles following the stopped vehicle by varying initial velocity, where (a) $v_{ini}=30\;\mathrm{km}/h$, (b) $v_{ini}=20\;\mathrm{km}/h$ and (c) $v_{ini}=10\;\mathrm{km}/h$. There are two vehicles colliding with their preceding vehicles when $v_{ini}=30\;\mathrm{km}/h$ (Figure 4a). By changing the initial velocity from $v_{ini}=30\;\mathrm{km}/h$ to $v_{ini}=20\;\mathrm{km}/h$, only one vehicle collides with its preceding vehicle (Figure 4b). No collision occurs when $v_{ini}=10\;\mathrm{km}/h$ (Figure 4c). Comparing with Figure 2b, it can be found that the decrease of initial velocity can reduce the number of crumpled vehicles in the proposed model for a two-lane traffic system.

**Figure 5:**
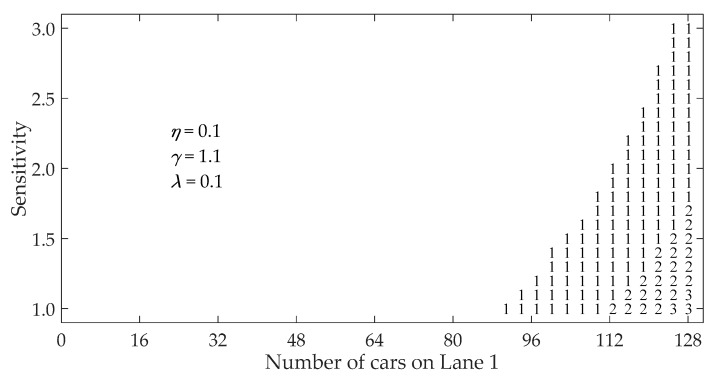
Region map (phase diagram) for the initial speed $v_{ini}=20\;\mathrm{km}/h$ with η=0.1. The numeral characters represent the number of crumpled vehicles, and the non-marked region represents no collision.

In order to examine the impact of initial velocity on region map, the simulation is carried out by changing the initial velocity from $v_{ini}=40\;\mathrm{km}/h$ to $v_{ini}=20\;\mathrm{km}/h$, as shown in Figure 5. Compared with Figure 3b, the collision region becomes small and the number of crumpled vehicles clearly reduces. It can be seen that initial velocity has a very significant impact on multiple-vehicle collision for the two-lane traffic flow in fog weather.

### 3.2. Small Perturbation Propagation

#### 3.2.1. Small Perturbation Propagation Under Different *η*

**Figure 6:**
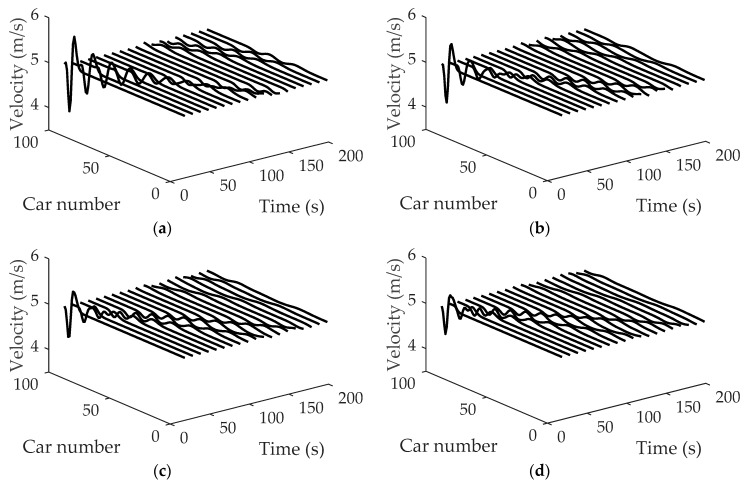
Space-time evolutions of velocity under different *η*: (**a**) η=0.1; (**b**) η=0.2; (**c**) η=0.3; (**d**) η=0.4, where α=1.5, λ=0.1, $\kappa_{1}=\kappa_{2}=0.8$.

Figure 6 shows the space-time evolutions of the velocity under different *η*, where (a) η=0.1, (b) η=0.2, (c) η=0.3 and (d) η=0.4. Comparing patterns (a), (b), (c) and (d) in Figure 6, it can be observed that with parameter *η* increasing, the amplitudes of velocity decrease. In addition, the traffic flow becomes more and more stable with the increase of parameter *η*, which indicates that drivers’ imitation of the neighboring lane’s vehicles has a positive influence on the stability of the traffic system.

**Figure 7:**
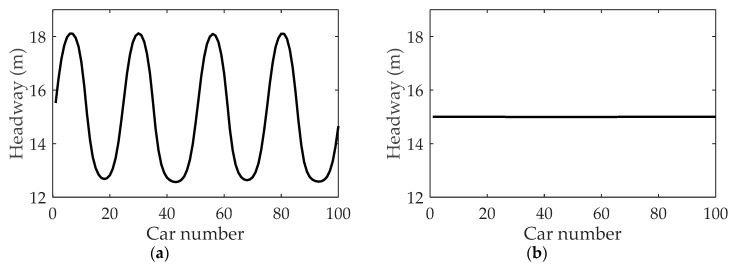
The headway under different *η*: (**a**) η=0.1; (**b**) η=0.2, where α=1.1, λ=0.1, $\kappa_{1}=\kappa_{2}=0.8$, t=8000 s.

The influence of the imitation phenomenon on headway is studied. When a small perturbation is added to the traffic system, Figure 7a indicates that the perturbation evolves into an unstable traffic flow after a sufficiently large time t=8000 s when η=0.1. However, Figure 7b depicts that the inhomogeneous flow has become stable as η increases to 0.2. Clearly, the results mean that the small perturbation can be absorbed under certain given conditions when considering the influence of the neighboring lane’s vehicles in fog weather.

#### 3.2.2. Small Perturbation Propagation Under Different *κ*_2_

**Figure 8:**
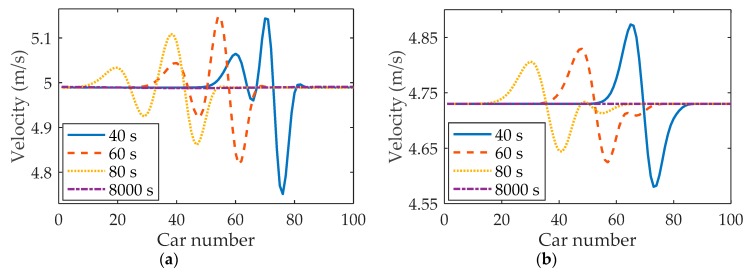
The velocity under different $\kappa_{2}$: (**a**) $\kappa_{2}=1.0$; (**b**) $\kappa_{2}=0.6$, where α=1.5, λ=0.1, η=0.2.

**Figure 9:**
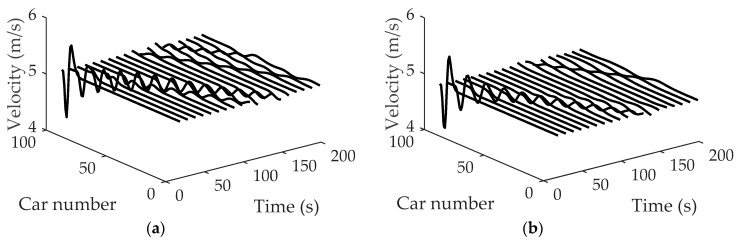
Space-time evolutions of velocity under different $\kappa_{2}$: (**a**) $\kappa_{2}=1.0$; (**b**) $\kappa_{2}=0.6$, where α=1.5, λ=0.1, η=0.2.

Numerical experiments are performed to test the influences of the neighboring lane’s vehicular velocity on traffic flow stability, acceleration and headway in fog weather. For comparison, parameter $\kappa_{2}$ is set to 0.6 and 1.0, respectively. Figure 8 and Figure 9 illustrate velocity profiles under different velocities ($\kappa_{2}$) in the neighboring lane. Comparing Figure 8a with Figure 8b, it is found that the velocity will decrease with the reduction of velocity in the neighboring lane. Moreover, the velocity fluctuation shown in Figure 9b is not as large as that of Figure 9a. Obviously, drivers’ velocity behaviors have a close relationship with the velocity in the neighboring lane, which is consistent with that in [41].

**Figure 10:**
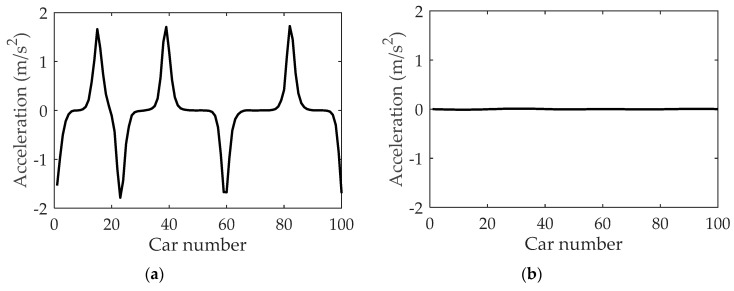
The acceleration under different $\kappa_{2}$: (**a**) $\kappa_{2}=1.0$; (**b**) $\kappa_{2}=0.6$, where α=1.1, λ=0.1, η=0.1, t=8000 s.

The velocity in the neighboring lane could also affect acceleration in fog weather. Figure 10 displays the acceleration profiles under different values of the parameter $\kappa_{2}$ after t=8000 s. For Figure 10a, where $\kappa_{2}=1.0$, there are obvious acceleration fluctuations. As the value of $\kappa_{2}$ decreases to 0.6 in Figure 10b, the acceleration approaches zero. It implies that lower vehicular velocity in the neighboring lane can reduce the magnitude of acceleration in the current lane. Namely, dangerous behaviors such as abrupt acceleration or deceleration can be reduced when vehicular velocity in the neighboring lane is lower.

**Figure 11:**
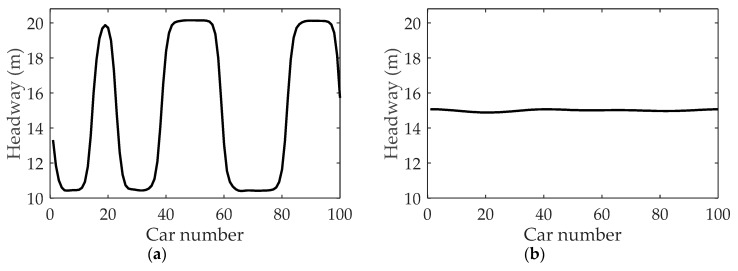
The headway under different $\kappa_{2}$: (**a**) $\kappa_{2}=1.0$; (**b**) $\kappa_{2}=0.6$, where α=1.1, λ=0.1, η=0.1, t=8000 s.

By merely changing parameter $\kappa_{2}$ from $\kappa_{2}=0.8$ in Figure 7a to $\kappa_{2}=1.0$, Figure 11a is obtained. Compared with Figure 7a, the fluctuation of headway in Figure 11a is aggravated as the vehicular velocity in the neighboring lane is increased in fog weather. Correspondingly, we conducted the simulation by changing $\kappa_{2}$ from $\kappa_{2}=0.8$ to $\kappa_{2}=0.6$. The headway profiles as $\kappa_{2}=0.6$ are shown in Figure 11b. It can be observed that due to the reduction of vehicular velocity in the neighboring lane, the fluctuation of headway in Figure 11a is not as large as that of Figure 7a and Figure 11a.

## 4. Discussion

Most previous studies have presented the influence factors of traffic flow in the same lane [10,11,12,13,14,15,16,17,18]. This paper, considering the imitation phenomenon from the neighboring lane’s vehicles in fog weather, proposes an extended car-following model to investigate whether or not the imitation phenomenon can influence traffic safety, as well as its influence mechanism. Compared to the previous single-lane car-following model [26], the new model in this study is extended to the two-lane situation, which is more practical and serves as a tool to study the effect of driver behavior on two-lane traffic safety.

In line with the previous studies [35,36], simulations of multiple-vehicle collision in this work demonstrate that the initial velocity of vehicles and the sensitivity of drivers as well as the density of traffic flow are all important factors affecting collisions. Furthermore, the results show that the imitation of a neighboring lane’s vehicles is able to reduce the number of collisions induced by a sudden stop in fog weather. Meanwhile, the region maps for the multiple-vehicle collision demonstrate that the collision region shrinks and the number of crumpled vehicles decreases with consideration of vehicles in the neighboring lane. All these findings illustrate that drivers’ imitation behavior of the neighboring lane’s vehicles is conducive to the improvement of traffic safety in fog weather.

In real traffic, various factors, for instance, sudden acceleration or deceleration, can be regarded as perturbations to the traffic flow. In the simulations of perturbation propagation of this study, a small perturbation (the headway deviation for vehicle 1) is set into the uniform traffic flow to investigate its effect on traffic velocity, acceleration and headway. Simulation results show that the perturbation can be absorbed under certain given conditions when considering the influence of the imitation phenomenon. It means that drivers’ imitation behavior contributes to the enhancement of stability of traffic flow in fog weather. Moreover, it is observed that the velocity of vehicles is influenced by the neighboring lane’s vehicles, which agrees well with the experimental results [5]. In addition, lower vehicular velocity in the neighboring lane can reduce the magnitude of acceleration and fluctuation of headway, which has a favorable influence on traffic safety. Conclusively, drivers’ imitation behavior of the neighboring lane’s vehicles is beneficial to traffic safety in fog weather.

The findings in this study can provide a theoretical reference for road safety management in fog weather. In particular, it can be applied to the development and implementation of multi-lane intermittent release measures. Shi and Tan found that intermittent release measures could reduce risk and energy consumption as well as exhaust emission [10,11,12]. They pointed out that an appropriate number of released vehicles should be selected when taking the single-lane intermittent release measures [10]. However, the multi-lane intermittent release measures were not discussed in their studies. In this work, drivers’ imitation behaviors of the neighboring lane’s vehicles are demonstrated to have an important effect on collision risk reduction. Accordingly, the multi-lane intermittent release measures appear to be more helpful to the improvement of traffic safety in fog weather.

In this work, the influence of imitation phenomenon on traffic safety in fog weather, which has not been extensively studied in the previous literature, is investigated based on the new model. The findings will be valuable for traffic management departments to develop multi-lane intermittent release measures, which helps to improve freeway traffic safety in fog weather. Nevertheless, some parameters of the new model should be further calibrated under different intermittent release measure conditions.

## 5. Conclusions

In fog weather, drivers will automatically imitate the behaviors demonstrated by other vehicles in the neighboring lane. In order to investigate whether or not the imitation phenomenon can influence traffic safety, this paper proposes a two-lane car-following model describing the effect of the imitation phenomenon of a neighboring lane’s vehicles in fog weather. It is found that the imitation of the neighboring lane’s vehicles is able to reduce the risk of collision in fog weather. A small perturbation added to the uniform traffic flow can be absorbed under certain given conditions when considering the influence of the imitation phenomenon. The findings of this work will be valuable for the development and implementation of multi-lane intermittent release measures, which are considered to have potential for traffic accident control and prevention. A direction to extend this work is to validate the numerical results using empirical data.

## Figures and Tables

**Table 1:** The average speed in Lane 1 under different visibility conditions in fog weather.

Visibility		24m	36m	48m
Average Speed (km/h)	Group 1	24.211	32.950	42.692
	Group 2	22.215	25.180	28.034
	Group 3	36.320	44.304	52.902
